# Schlemm’s Canal and Trabecular Meshwork in Eyes with Primary Open Angle Glaucoma: A Comparative Study Using High-Frequency Ultrasound Biomicroscopy

**DOI:** 10.1371/journal.pone.0145824

**Published:** 2016-01-04

**Authors:** Xiaoqin Yan, Mu Li, Zhiqi Chen, Ying Zhu, Yinwei Song, Hong Zhang

**Affiliations:** Department of Ophthalmology, Tongji Hospital, Tongji Medical College, Huazhong University of Science and Technology, Wuhan, China; Duke University, UNITED STATES

## Abstract

We investigated *in vivo* changes in Schlemm’s canal and the trabecular meshwork in eyes with primary open angle glaucoma (POAG). Relationships between Schlemm’s canal diameter, trabecular meshwork thickness, and intraocular pressure (IOP) were examined. Forty POAG patients and 40 normal individuals underwent 80-MHz Ultrasound Biomicroscopy examinations. The Schlemm’s canal and trabecular meshwork were imaged in superior, inferior, nasal and temporal regions. Normal individuals had an observable Schlemm’s canal in 80.3% of sections, a meridional canal diameter of 233.0±34.5 μm, a coronal diameter of 44.5±12.6 μm and a trabecular meshwork thickness of 103.9±11.1 μm, in POAG patients, Schlemm’s canal was observable in 53.1% of sections, a meridional canal diameter of 195.6±31.3 μm, a coronal diameter of 35.7±8.0 μm, and a trabecular meshwork thickness of 88.3±13.2 μm, which significantly differed from normal (both p <0.001). Coronal canal diameter (r = -0.623, p < 0.001) and trabecular meshwork thickness (r = -0.663, p < 0.001) were negatively correlated with IOP, but meridional canal diameter was not (r = -0.160, p = 0.156). Schlemm’s canal was observable in 50.5% and 56.6% of POAG patients with normal (<21 mmHg) and elevated (>21 mmHg) IOP, respectively (χ = 1.159, p = 0.282). Coronal canal diameter was significantly lower in the elevated IOP group (32.6±4.9 μm) than in the normal IOP group (35.7±8.0 μm, p < 0.001). This was also true of trabecular meshwork thickness (81.9±10.0 μm vs. 97.1±12.0 μm, p < 0.001). In conclusion, eyes with POAG had fewer sections with an observable Schlemm’s canal. Canal diameter and trabecular meshwork thickness were also lower than normal in POAG patients. Schlemm’s canal coronal diameter and trabecular meshwork thickness were negatively correlated with IOP.

## Introduction

Glaucoma is a leading cause of irreversible blindness worldwide [1]. It is characterized by progressive visual field defects and optic atrophy. The most important risk factor for glaucoma is elevated intraocular pressure (IOP) [2], with aqueous outflow cycling playing an important role in IOP regulation. After being secreted by the ciliary body, aqueous humor arrives in anterior chamber, then through trabecular meshwork (TM) draining into Schlemm’s canal (SC), and then next to the collector channels and the intrascleral plexus, entering the episcleral veins which finally back to the blood circulation to maintain a dynamic balance [3]. To maintain IOP within the normal physiological range of 10-21mmHg, the aqueous humor outflow rate through the conventional TM pathway must equal the aqueous humor production rate. Maepea and Bill [4] showed that, in monkey eyes, nearly 90% of the outflow resistance was located in the sub-endothelial region of SC. Additionally, Grant [5] concluded that approximately 75% of the resistance to flow in enucleated human eyes was located internal to SC, within the TM, when perfusion pressure was 25 mmHg. Following complete trabeculectomy [6], 71% of the outflow resistance was eliminated at a perfusion pressure of 25 mmHg, but only 49% of the resistance was eliminated at a normal perfusion pressure of 7 mmHg. Gong et al. [7] reported that one third to one half of the outflow resistance is located distal to the inner wall of SC. These results suggested that pressure-dependent changes in outflow resistance occurred in the TM, SC, and distal to SC [4–7]. Abnormal aqueous humor outflow pathway resistance can result in excess aqueous humor and elevated IOP.

In 1973, an opinion was formed based on the observations of Jonestone and Grant [8] regarding the relationship between SC and IOP. They showed that acute elevation of IOP might cause the SC to collapse and the TM to compression, both of which would further increase resistance of the aqueous outflow pathway and begin a vicious cycle of progressively increasing IOP. The major aqueous outflow resistance located in the JCT region and the inner wall of Schlemm’s canal [9]. In 1996, a clinical study revealed that SC cross-sectional area, perimeter, and inner wall length were smaller in eyes with POAG than in normal eyes. It also demonstrated the reduction in SC dimensions accounting for approximately 50% of the decrease in aqueous outflow facility in POAG eyes, which suggested that a significant correlation between SC size and aqueous outflow capacity, [10]. Based on above findings, surgical procedures on SC (e.g., canaloplasty, iStent and Eypass implantation) and the TM (e.g., trabectome) have been launched worldwide. The features of SC and the TM have played an important role in the development of these new techniques. Bull et al. [11] showed that canaloplasty successfully reduced IOP from 23.0 ± 4.3 mmHg before surgery to 15.1 ± 3.1 mmHg three years after surgery in eyes with POAG, demonstrating that changes in SC could affect IOP. The effects of changes in the TM have also been shown, with trabectome now being applied in some developed countries. This procedure generally results in a mean IOP reduction of approximately 30–40% [12, 13].

Previous conclusions about SC and the TM were made *in vitro*, not *in vivo*, studies than examined ocular structures using micro-CT, light microscopy, or electron microscopy. To better understand these results and to acquire a more theoretical foundation for new surgical techniques, the physiological activity of the SC and TM need to be directly examined *in vivo* studies.

Advancements in medical imaging make it convenient and possible to study SC and the TM *in vivo*. Asrani et al. [14] were the first to use Fourier-domain optical coherence tomography (OCT) to visualize SC and the TM, allowing them to measure SC and TM size. The high-density OCT was more recently used to show that acute IOP elevations in healthy eyes resulted in a reduced SC cross-sectional area [15]. In addition, Hong et al. [16] used spectral-domain OCT to show that SC size was significantly different in eyes with and without POAG and that SC area was negatively correlated with IOP. The above studies showed that the *in vivo* research on the SC and TM was possible, but these studies were limited in that they only examined the SC and TM in one or two quadrants. Therefore, the data may not represent overall changes in these structures. Using 80-MHz ultrasound biomicroscopy, a noninvasive, real-time, dynamic, continuous observation technique, high resolution *in vivo* images of the SC can be obtained. In 2010, Irshad [17] prospectively measured *in vivo* variations in SC diameter and location using 80-MHz ultrasound biomicroscopy, with measurements taken at 12’o clock in 94 subjects that did or did not have glaucoma. However, the population examined had wide variations in race and surgical history [17]. No previous study has examined changes in SC and TM features in patients with POAG using 80-MHz ultrasound biomicroscopy, which can continuously and dynamically observe and record anterior chamber structures in detail. Owing to new theories regarding the role of TM and SC changes in POAG, new surgical techniques specifically treating TM and SC abnormalities were developed. New methods to image SC and the TM allowed these structures to be thoroughly studied to better understand this physiological channel. The purpose of the current study was to evaluate and compare SC and TM parameters in normal individuals and in patients with POAG using 80-MHz ultrasound biomicroscopy. Main outcome measurements included the percentage of sections with an observable SC, SC diameter, and TM thickness.

## Materials and Methods

This study was approved by the ethics committee of the Tongji Hospital, part of the Medical College of Huazhong University of Science and Technology Institute. All study conduct adhered to the tenets of the Declaration of Helsinki and all subjects provided written informed consent to participate in the study.

### Subjects

This observational, comparative study included 44 patients with POAG who visited the Department of Ophthalmology, Tongji Hospital, Tongji medical college, Huazhong University of Science and Technology, between March and May of 2014. Additionally, 42 age- and gender-matched normal subjects were enrolled into a control group. Subjects were included in the POAG group if all of the following were true: (1) at least 18 years of age, (2) cup-to-disc (C/D) ratio ≥ 0.6 with an interocular C/D ratio difference ≥ 0.2, (3) retinal nerve fiber layer defect was present, (4) glaucomatous visual field defects corresponding to optic nerve changes were present, (5) normal anterior chamber depth with an open angle, and (6) refractive error between +3.0 and -6.0 diopters (D). Patients who had prior ocular surgeries or a history of eye disease (except for POAG) were excluded from participation. Patients with systemic disease were also excluded. Normal subjects were included if all of the following were true: (1) at least 18 years of age, (2) normal fundus, (3) normal visual field, (4) normal anterior chamber depth with an open angle, and (5) a refractive error between +3.0 and -6.0 D. Potential control patients were excluded from participation if they had a family history of glaucoma, a history of ophthalmic disease or surgery, or systemic disease.

Subjects with POAG were divided into two groups based on IOP measurements of each eye. If IOP was >21 mmHg (24 right eyes, 22 left eyes) the eye was placed in the elevated IOP subgroup and if IOP was <21 mmHg (16 right eyes, 18 left eyes) the eye was placed in the normal IOP subgroup and it was assumed that the subject used anti-glaucoma eye drops.

### Study Examinations

All subjects underwent a comprehensive ophthalmologic examination, which included measurement of visual acuity, refractive error, IOP (non-contact tonometer), and axial length (AL). Slit-lamp examination, gonioscopy, 80-MHz ultrasound biomicroscopy (iScience Interventional, Inc., Menlo Park, CA), optic nerve and fundus photography, and visual field testing (Humphery perimetry with the 30–2 threshold test protocol). Anterior (Visante OCT) and posterior (SD-OCT Heidelberg Engineering GmbH, Heidelberg, Germany) segment OCT were also performed to measure central corneal thickness (CCT) and retinal nerve fiber layer thickness, respectively.

### Schlemm’s Canal and Trabecular Meshwork Biomicroscopy Measurements

**Fig 1:**
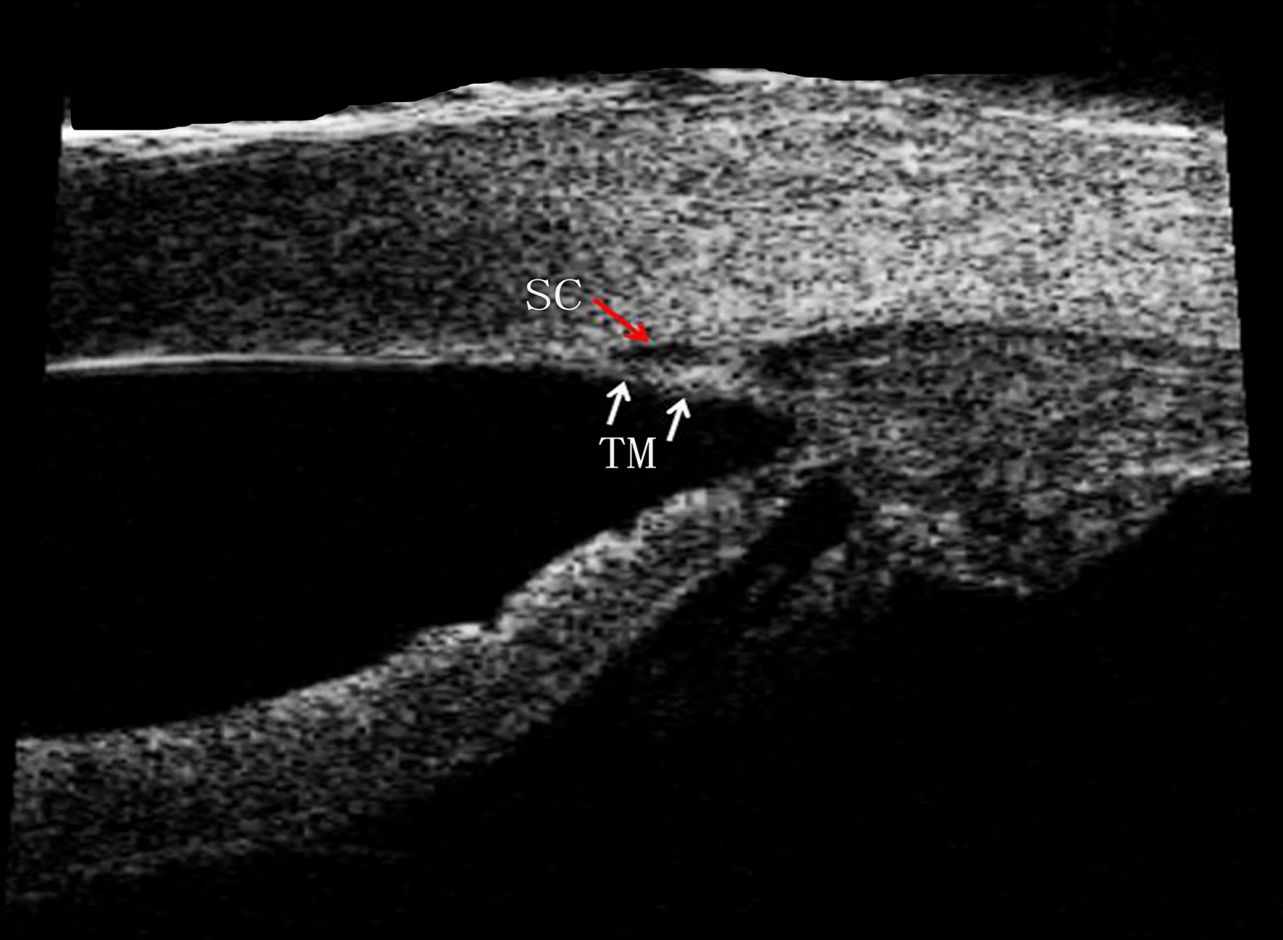
An 80-MHz Ultrasound Biomicroscopy Image of Schlemm’s Canal and the Trabecular Meshwork in a Normal Individual. Schlemm’s canal (red arrow) and the trabecular (white arrow) are apparent in the image.

Imaging of SC and the TM was conducted by the same experienced technician using the iUltrasound imaging system. The observer remained masked to patient group assignment. Images were obtained using the following iUltrsound system settings: transducer frequency = 80-MHz, axial resolution = 25 μm, lateral resolution = 50 μm, electronic resolution = 10 μm, caliper positioning limit = 10μm, tissue penetration depth = 2 mm, scan rate = 7 frames/s, and imaging window size = 4.0 × 4.0 mm. Before iUltrasound measurements were made, eyes were anesthetized with topical 1% oxybuprocaine and a low-viscosity ultrasound gel was placed on the ocular surface. The iUltrasound probe was directly placed on the eye and SC and TM parameters were directly measured from images using the built-in electronic and digital caliper application of the iUltraSound imaging system. Each eye had measurements taken at the 12, 3, 6 and 9 o’clock positions (Fig 1).

**Fig 2:**
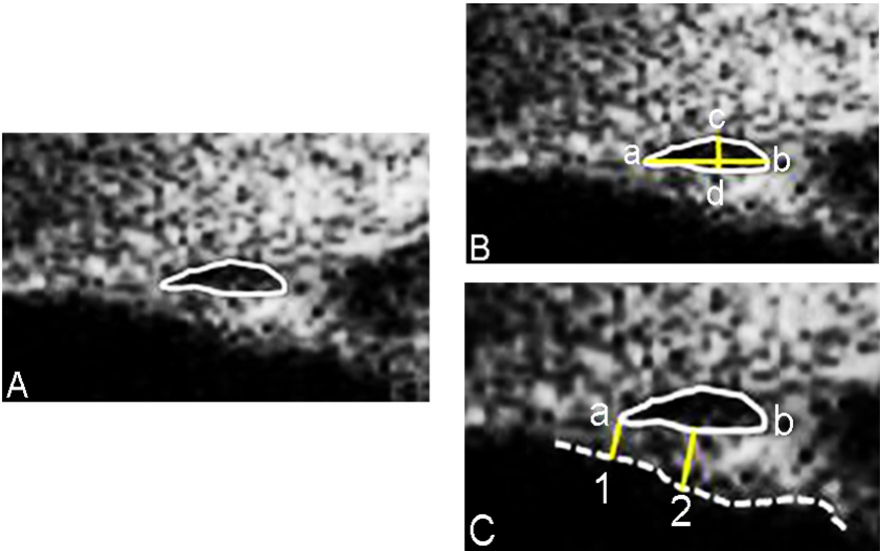
Example of Schlemm’s Canal and Trabecular Meshwork Measurements Made Using the iUltrasound Imaging System. The black oval space shows Schelmm’s canal (SC). The meridional diameter of SC was measured from the anterior (a) to the posterior (b) end point of SC. To measure the coronal diameter of SC, we drew a vertical line across the canal to get two intersection points (c and d). The maximum distance between c and d was taken as the coronal diameter of SC. Lines 1, 2 indicated where trabecular meshwork thickness was measured and the dotted line shows the meshwork inner layer.

The SC was defined as observable when a thin, black, lucent space was found in two images. Optimum image contrast and magnification and previous histology manifestations were subjectively defined to maximize SC visualization. The percentage of sections with an observable SC (eyes with a completely observable SC/total number of eyes × 100), the longest SC meridional diameter (diameter of the white oval space measured from the posterior to the anterior SC end point), and the SC coronal diameter (maximum distance from the inner to the outer wall of SC) were measured. To determine SC coronal diameter, we manually drew a vertical line across the canal to obtain two intersection points (points c and d). We then measured the maximum distance between c and d. The TM thickness was calculated as the average of two measurements made at the anterior end point of SC and halfway down the SC. The posterior end point of SC was not chosen, the measurement of TM at this region might not truly represent the thickness of TM itself, but the measurement of ciliary muscle behind the sclera spur [18–20]. Each TM thickness measurement was made perpendicular to the inner layer of the meshwork (Fig 2).

### Statistical Analyses

All analyses were performed using the SPSS software package version 19.0. Data were presented as mean ± standard deviation where applicable. The Mann–Whitney U test, the Kruskal-Wallis H test, and the Chi-square test were used for comparing differences between groups. Nonparametric Spearman correlation analyses were performed to statistically examine the relationships between IOP and SC and TM parameters. All tests were two-tailed and statistical significance was defined as p < 0.05.

## Results

A total of 44 POAG patients and 42 normal individuals were enrolled in this study. Of these, 4 POAG patients and 2 normal individuals were excluded because of poor image quality. Therefore, 40 POAG patients (26 men, 14 women) and 40 normal individuals (26 men, 14 women) were ultimately included in analyses. Both eyes of all 80 participants (160 eyes) were included in the study. Mean subject age was 40.8 ± 12.4 years in the normal group and 40.7 ± 13.9 years in the POAG group, a slight difference that was not statistically significant (p = 0.795). Mean IOP was 15.6 ± 2.6 mmHg in both the right and left eyes of the normal group and 26.2 ± 10.9 and 25.2 ± 9.4 mmHg in the right and left eyes of the POAG group, respectively (both p < 0.001). There were no significant differences between the normal and POAG groups in refractive error, CCT, and AL, as summarized in Table 1.

**Table 1:** Subject and Ocular Characteristics.

	Groups		
	Normal	POAG	p-value ^a^
**Male/Female**	26/14	26/14	
**Age (years)**	40.8 **±** 12.4	40.7 **±** 13.9	0.795
**IOP-Right (mmHg)**	15.6 **±** 2.6	26.2 ± 10.9	<0.001
**IOP-Left (mmHg)**	15.6 **±** 2.6	25.2 **±** 9.4	<0.001
**CCT-Right (μm)**	536.1 ± 30.0	540.8 ± 30.4	0.554
**CCT-Left (μm)**	535.0 ± 31.3	540.3 ± 30.8	0.422
**AL-Right (mm)**	23.7 ± 1.2	24.1 ± 0.9	0.108
**AL-Left (mm)**	23.7 ± 1.1	24.0 ± 0.8	0.149
**Refraction (D)-Right**	-0.96 ± 1.87	-1.32 ± 2.10	0.699
**Refraction (D)-Left**	-0.88 ± 1.76	-1.24 ± 1.91	0.633
**MD-Right (dB)**	-0.74 ± 1.61	-10.8 ± 11.3	<0.001
**MD-Left (dB)**	-0.65 ± 1.42	-11.7 ± 11.5	<0.001
**Best-Corrected VA-Right**	1.06 ± 0.15	0.95 ± 0.27	0.027
**Best-Corrected VA-Left**	1.01 ± 0.19	0.95 ± 0.28	0.225

Data were presented as mean ± standard deviation. CCT = central corneal thickness, IOP = intraocular pressure, AL = axial length, D = diopters, MD = mean deviation, VA = visual acuity, POAG = primary open angle glaucoma.

^a^ Statistical significance of differences tested with a Mann–Whitney U test.

When subjects with POAG were divided into subgroups based on IOP, those in the elevated IOP group had a mean IOP of 32.3 ± 10.0 mmHg in the right eye and 31.5 ± 8.3 mmHg in the left eye. In subjects with normal IOP, mean IOP was 17.0 ± 2.6 mmHg in the right eye and 17.6 ± 2.5 mmHg in the left eye.

### Schlemm’s Canal and Trabecular Meshwork Parameters

Table 2 shows the percentage of sections with an observable SC in each study group. The SC was observable significantly less often in eyes with POAG (53.1%) than in normal eyes (80.3%, χ = 53.261, p < 0.001). Eyes with POAG and elevated IOP had an observable SC in 50.5% of sections and eyes with POAG and normal IOP had an observable SC in 56.6% of sections, a slight difference that was not statistically significant (χ = 1.159, p = 0.282). However, both values were significantly lower than in normal eyes (χ = 35.389, 21.213, both p < 0.001, Table 3).

**Table 2:** Proportion of Eyes with an Observable Schlemm’s Canal.

	Eyes with observable SC			
	Normal	POAG	χ	p-value
**Right eyes, n (%)**	129/160 (80.6)	88/160 (55.0)	24.067	<0.001
**Superior, n (%)**	35/40 (87.5)	23/40 (57.5)	9.028	0.003
**Nasal, n (%)**	30/40 (75.0)	18/40 (45.0)	7.500	0.006
**Inferior, n (%)**	31/40 (77.5)	23/40 (57.5)	3.647	0.056
**Temporal, n (%)**	33/40 (82.5)	24/40 (60.0)	4.943	0.026
**p-value** ^**a**^	0.501	0.528		
**Left eyes, n (%)**	128/160 (80.0)	82/160 (51.3)	29.313	<0.001
**Superior, n (%)**	32/40 (80.0)	21/40 (52.5)	6.765	0.009
**Nasal, n (%)**	31/40 (77.5)	19/40 (47.5)	7.680	0.006
**Inferior, n (%)**	32/40 (80.0)	22/40 (55.0)	5.698	0.017
**Temporal, n (%)**	33/40 (82.5)	20/40 (50.0)	9.448	0.002
**p-value** ^**a**^	0.958	0.919		
**Total, n (%)**	257/320 (80.3)	170/320 (53.1)	53.261	<0.001

Statistical comparisons made using chi-square tests. SC = Schlemm’s canal, POAG = primary open angle glaucoma.

^a^ difference among superior, nasal, inferior and temporal sections.

**Table 3:** Proportion of Eyes with Primary Open Angle Glaucoma That Had an Observable Schlemm’s Canal.

	Observable Schlemm’s Canal					
		POAG				
	Normal	IOP>21mmHg	IOP<21mmHg	p-value ^a^	p-value ^b^	p-value ^c^
**Right eyes, n (%)**	65/80 (81.3)	49/96 (51.0)	39/64 (60.9)	<0.001	0.007	0.218
**Left eyes, n (%)**	65/80 (81.3)	44/88 (50.0)	38/72(52.8)	<0.001	<0.001	0.727
**Total, n (%)**	130/160 (81.3)	93/184 (50.5)	77/136 (56.62)	<0.001	<0.001	0.282
**p-value** ^**d**^	1.000	0.888	0.338			

All statistical comparisons were performed using a chi-square test. POAG = primary open angle glaucoma, IOP = intraocular pressure.

^a^ comparison between Normal and POAG (IOP > 21 mmHg) groups.

^b^ comparison between Normal and POAG (IOP < 21 mmHg) groups.

^c^ comparison between POAG groups.

^d^ comparison between right eyes and left eyes.

Patients with POAG had a significantly smaller SC meridional diameter (233.0 ± 34.5 μm vs. 195.6 ± 31.3 μm, p < 0.001), SC coronal diameter (44.5 ± 12.6 μm vs. 35.7 ± 8.0 μm, P < 0.001), and TM thickness (103.9 **±** 11.1 μm vs. 88.3 **±** 13.2 μm, P < 0.001; Tables 4 and 5) than normal individuals.

**Table 4:** Characteristics of Schlemm’s Canal.

	Groups		
	Normal	POAG	p-value
**Meridional diameter (μm)**			
**Right eyes**	234.4 **±** 30.4	194.5 **±** 27.2	<0.001
**Superior**	235.4 **±** 30.4	191.7 **±** 32.3	<0.001
**Nasal**	234.0 **±** 37.0	196.7 **±** 25.0	0.001
**Inferior**	236.1 **±** 33.4	196.1 **±** 20.4	<0.001
**Temporal**	232.1 **±** 34.6	194.2 **±** 30.5	<0.001
**P-Value** ^**a**^	0.924	0.916	
**Left eyes**	231.6 **±** 35.7	196.7 **±** 35.2	<0.001
**Superior**	225.3 **±** 33.0	197.1 **±** 31.0	0.002
**Nasal**	230.3 **±** 40.7	191.1 **±** 34.3	0.001
**Inferior**	233.4 **±** 37.0	196.4 **±** 32.6	0.001
**Temporal**	237.3 **±** 32.1	202.0 **±** 44.0	0.004
**p-value** ^**a**^	0.681	0.816	
**Average**	233.0 **±** 34.5	195.6 **±** 31.3	<0.001
**Coronal diameter (μm)**			
**Right eyes**	44.6 **±** 8.1	35.1 **±** 5.7	<0.001
**Superior**	44.0 **±** 12.9	35.2 **±** 6.7	0.006
**Nasal**	44.0**±** 13.5	35.6 **±** 8.6	0.032
**Inferior**	44.5 **±** 10.9	34.8 **±** 6.7	0.001
**Temporal**	44.8 **±** 12.5	36.3 **±**11.3	0.004
**p-value** ^**a**^	0.960	0.944	
**Left eyes**	44.7**±** 7.6	35.5 **±** 5.9	<0.001
**Superior**	44.4 **±** 12.9	35.7**±** 7.5	0.014
**Nasal**	44.5**±** 11.2	36.3 **±** 7.6	0.01
**Inferior**	43.1 **±** 13.3	35.5 **±** 6.7	0.03
**Temporal**	47.0 **±** 13.6	36.5 **±** 8.8	0.003
**p-value** ^**a**^	0.675	0.990	
**Average**	44.5 **±** 12.6	35.7 **±** 8.0	<0.001

Mann–Whitney U test and Kruskal-Wallis H tests were used to test statistical significance. POAG = primary open angle glaucoma.

^a^ difference among superior, nasal, inferior and temporal sections.

**Table 5:** Trabecular Meshwork Thickness.

	TM thickness (μm)		
	Normal	POAG	p-value
**Right eyes**	105.4 **±** 10.0	86.1 **±** 13.0	<0.001
**Superior**	105.1 **±** 17.5	85.9**±** 18.2	<0.001
**Nasal**	106.5 **±** 13.1	86.4 **±** 16.2	<0.001
**Inferior**	103.4 **±** 20.2	86.5 **±** 16.1	0.004
**Temporal**	107.9 **±** 13.5	87.7 **±** 14.6	<0.001
**p-value** ^**a**^	0.702	0.966	
**Left eyes**	102.3 **±** 12.1	90.6 **±** 13.2	<0.001
**Superior**	101.4 **±** 14.8	91.0 **±** 11.1	0.005
**Nasal**	106.1 **±** 18.7	91.8 **±** 17.7	0.011
**Inferior**	99.2 **±** 18.3	90.2 **±** 13.6	0.06
**Temporal**	104.8 **±** 17.1	87.9 **±** 17.6	0.001
**p-value** ^**a**^	0.372	0.863	
**Average**	103.9 **±** 11.1	88.3 **±** 13.2	<0.001

Mann–Whitney U test and Kruskal-Wallis H tests were used to test statistical significance. TM = trabecular meshwork, POAG = primary open angle glaucoma.

^a^ difference among superior, nasal, inferior and temporal sections.

**Fig 3:**
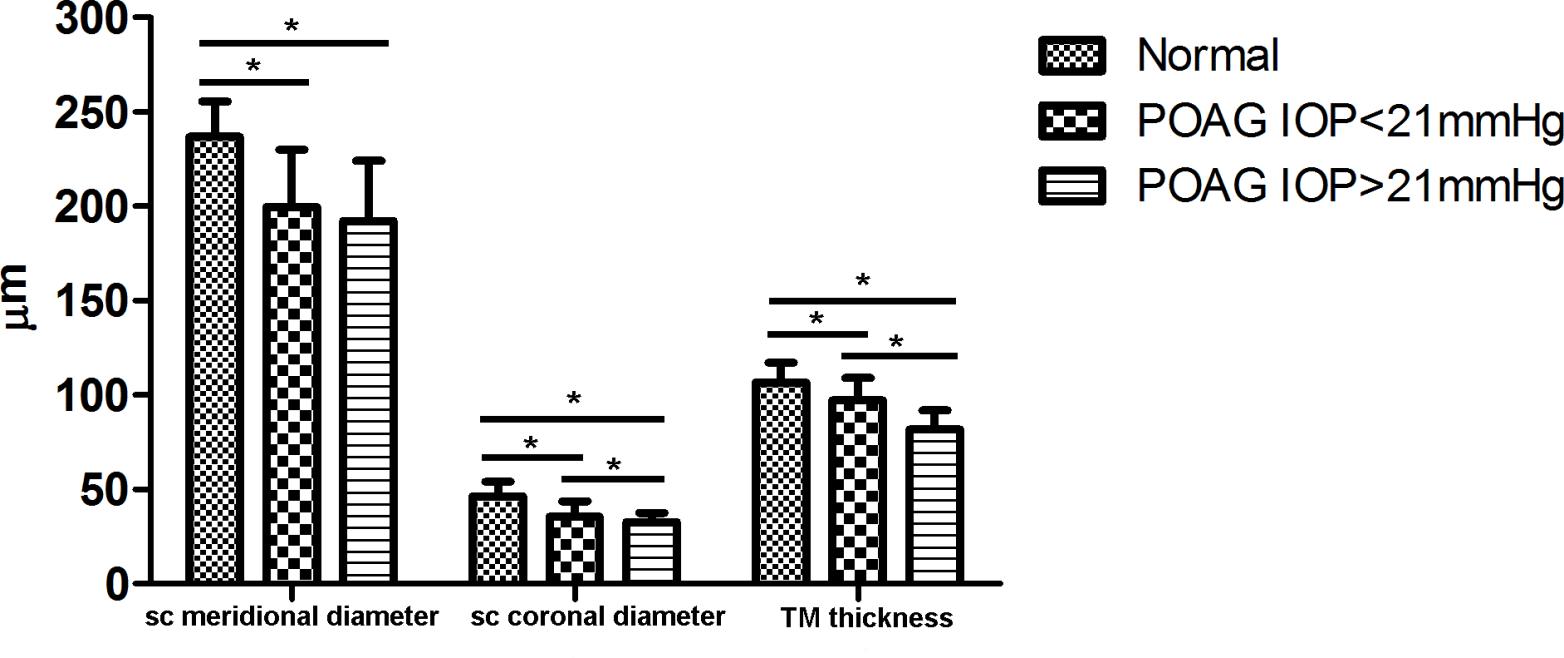
Schlemm’s Canal Meridional and Coronal Diameter and Trabecular Meshwork Thickness in Patients with Primary Open Angle Glaucoma. Schlemm’s canal (SC) and trabecular meshwork (TM) measurements for patients with primary open angle glaucoma (POAG) and normal IOP (< 21 mmHg) and patients with POAG and elevated IOP (> 21 mmHg). *indicates a statistically significant difference.

The POAG subjects with elevated IOP had a smaller SC coronal diameter (32.6 **±** 4.9 μm) than POAG subjects with normal IOP (35.7 ± 8.0 μm, p < 0.001). This was also true of TM thickness (81.8 ± 10.0 μm vs. 97.1 ± 12.0 μm, p < 0.001), but not of SC meridional diameter (192.2**±**31.8 μm [elevated IOP] vs. 199.7**±**30.3 μm [normal IOP], p = 0.242; Fig 3). The same was true when the right and left eyes were examined individually. In normal individuals, there was no significant difference in the percentage of sections with an observable SC, SC diameter, or TM thickness between the right and left eyes. As a reminder, SC diameter and TM thickness were only measured using images in which SC was completely observable.

**Fig 4:**
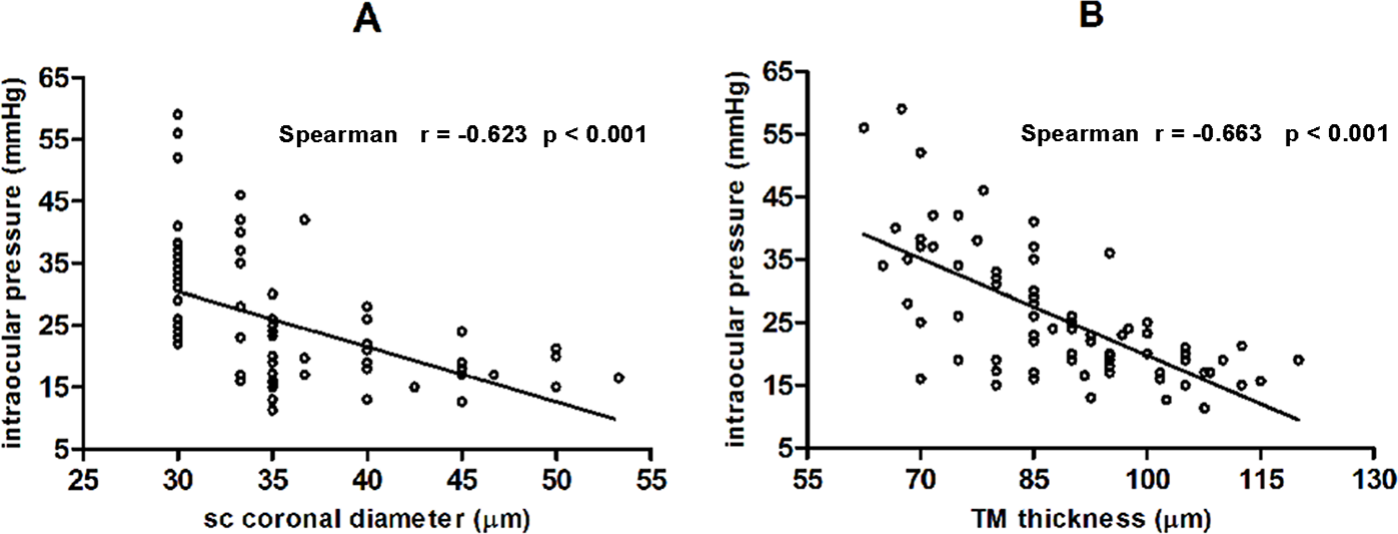
Correlations between Schlemm’s Canal Coronal Diameter, Trabecular Meshwork Thickness, and Intraocular Pressure. Schlemm’s canal coronal diameter and trabecular meshwork thickness were both significantly and negatively correlated with intraocular pressure in patients with primary open angle glaucoma.

Additionally, SC coronal diameter (r = -0.623, p < 0.001) and TM thickness (r = -0.663, p <0.001) were significantly and negatively correlated with IOP in the POAG group. However, SC meridional diameter and IOP were not significantly correlated (r = -0.160, p = 0.156; Fig 4).

## Discussion

This study investigated SC and the TM, two major structures in the aqueous outflow pathway. We first compared SC and TM morphology in normal individuals and POAG patients using *in vivo* measurements. Measurements were made using comprehensive and accurate SC and TM images obtained with 80-MHz ultrasound biomicroscopy. We examined four regional (12, 3, 6 and 9 o’clock) SC and TM parameters.

In our current study, the SC was observable in 53.1% of eyes with POAG, which was significantly lower than in the normal group (80.3%). In contrast, Hong et al. [16] previously used spectral-domain OCT to image SC and found that it was observable in 80% of eyes with POAG. We speculate that this might result from several reasons. First, OCT axial resolution was 6 μm, but the axial resolution of the 80-MHz transducer was only 25 μm, leading unable to identify a markedly narrowed SC. We found that the percentage of POAG eyes with an observable SC on high-frequency ultrasound biomicroscopy was slightly, but not significantly, higher when IOP was normal (56.6%) than when IOP was elevated (50.5%). If the axial resolution would be higher, different SC closure rate might be detected between two IOP subgroups. Second, observations with ultrasound biomicroscopy were continuous and dynamic, but observations with spectral-domain OCT were static. Third, elevated IOP was not the only factor responsible for SC collapse in eyes with POAG and SC anatomical variations and other factors regulating SC were likely involved. Swain et al. [19] demonstrated that scleral spurs were important for supporting and maintaining SC patency. Mean scleral spur length, maximum scleral spur width, and scleral spur posterior movement upon ciliary muscle contraction were smaller in eyes with POAG eyes than in normal eyes. They also showed that eyes with a shorter scleral spur posterior movement had a higher incidence of SC collapse [19]. The ciliary muscles, controlled by parasympathetic nerve activity, might pull on the conventional aqueous humor outflow pathway and prevent SC collapse when contracting [21]. In addition, noradrenergic fibers and β2-adrenergic receptors were present in SC [22–23]. It may be that POAG patients have abnormalities in these or other unknown SC regulating factors.

The anatomy of SC has been studied for a long time. In 1971, Hoffman et al. [24] revealed that SC diameter was approximately 190–350 μm in cadaver eyes, as measured with electron microscopy images. Since that time, SC has been studied using histology techniques in fixed tissue, which was not representative of the physiological situation. Advances in imaging technology allowed Usui et al. [25] to use Fourier-domain OCT to measure SC length, which was 347.2 ± 42.3 μm, in unfixed, enucleated human eyes. Additionally, Shi et al. [26] reported an SC meridional diameter of 266.96 ± 49.55 μm in normal individuals. Our findings were consistent with these previous studies.

It was well-known that the SC played a role in POAG. Early in the 18th century, researchers discovered that SC was a thin-walled, blood-filled canal located in the scleral sulcus [27]. Grieshaber et al. [28] assessed SC status by observing blood reflux from collector channels to SC, with a lack of refluxed blood indicating complete canal collapse. A negative correlation between reflux and IOP was found in eyes with POAG (regular filling when mean IOP was 30 mmHg, patchy filling when mean IOP was 40 mmHg, and no filling when mean IOP was ≥ 50 mmHg) [28]. Elevated IOP was not the only reason for a lack of blood reflux into SC. Aqueous humor drainage preferentially occurs near collector channels (CCs) [7, 29]. CC ostia obstructed by TM herniations when IOP increasing and might lead to the SC blood reflux absence from the episcleral venous system into SC, which could help evaluate the patency of the distal outflow pathway [30, 31]. With IOP elevation, TM herniated into CC ostia, the percentage of ostia obstructed by herniations was increased (15.6% ± 6.5% at 7 mm Hg, 46.4% ± 3.9% at 15mmHg, 95% ± 2.3% at 30 mmHg, 100% at 45 mm Hg) and effective filtration length (The ratio of the filtration length of the inner wall exhibiting tracer labeling to the total length of the inner wall of the aqueous plexus) was decreased [30]. Gong et al. [31] indicated that weakened and interrupted blood reflux into SC from CCs was consistent with a decrease in the number of quadrants with fluorescein egress into episcleral veins via CCs and that blockage of CC ostia existed *in vivo* in POAG. Using three-dimensional micro-computed tomography (CT) reconstruction of the SC and collector channel, a recent study showed that SC was more discontinuous and had fewer anastomosing channels in eyes with POAG than in normal eyes at the same perfusion pressure [32]. Using spectral-domain OCT, Kagemann et al. [15] reported that acute IOP elevation (12.5 to 36.1 mm Hg) in healthy eyes significantly reduced SC cross-sectional area. Imaging revealed that SC compression occurred because of inner wall movement towards the outer wall [15]. Shi et al. [26] used swept source OCT to measure SC meridional diameter, which was significantly larger in normal individuals (272.83 ± 49.39 μm) than in patients with POAG (190.91 ± 46.47 μm) [26]. Wang et al. [33] also used swept source OCT to measure SC circumference, area, and long diameter in POAG patients. All parameters were smaller in POAG patients than in normal individuals [33]. Our results were in agreement with these previous studies and we found that both meridional and coronal SC diameter were significantly smaller in eyes with POAG than in normal eyes. We also found that the SC coronal, but not meridional diameter was correlated with IOP and was smaller in eyes with POAG and elevated IOP than in eyes with POAG and normal IOP. We speculated that SC meridional diameter was only one indicator and did not reflect SC collapse.

The TM consists of anterior and posterior regions. The anterior portion of TM is known as “nonfiltering” meshwork which is not adjacent to SC and has no aqueous humor filtration, the posterior portion of TM is known as “filtering” meshwork which leads to SC. Yang et al. [20] reported that outflow facility increased after perfusion with Y27632 (a Rho-kinase inhibitor), the increase in outflow facility correlated positively with an increase in effective filtration length, which was associated with expansion in JCT. Meanwhile, TM and JCT thickness was thicker in high-tracer regions than low-tracer regions in human eyes. They postulated that the increase in outflow facility by Y27632 due to the effect of increased TM and JCT expansion, in which JCT expansion played the leading role. Besides, previous studies have confirmed that TM could be observed and measured *in vivo* with medical imaging [25, 34]. Therefore, we measured the thickness of TM below the SC (“filtering” meshwork) and compared the TM thickness between POAG patients and normal individuals with 80-MHz ultrasound biomicroscopy. The TM thickness was smaller in glaucoma patients (88.3 ± 13.2 μm) than in normal individuals (103.9±11.1 μm) and was negatively correlated with IOP significantly in this study. The direct effect of increased IOP, TM fibrosis, TM stiffness, and TM atrophy could all lead to TM alternations. Grant et al. [8] found that the TM might compress with acute elevation in IOP. Filla et al. [35] reported that increased extracellular matrix expression and deposit in the TM resulted in an increase in IOP. Additionally, a number of studies have shown that fibrous granular material deposition and increased TM electron density contributed to increasing outflow system resistance in eyes with glaucoma [36–41]. Unfortunately, the composition of the extracellular matrix remained unclear, but collagen fibers are a main extracellular matrix component and play an important role in aqueous outflow [42]. Types I, II, III, and VI collagen have been found in the TM, but the amount of type VI collagen increased in eyes with glaucoma [43]. Millard et al. [44] showed that the amount of type I collagen increased in the TM. The dysregulation of the extracellular matrix caused fibrosis and increased stiffness of the TM. TM stiffness that could influence the extent of deformation of TM and the inner wall of SC [45–47]. Using atomic force microscopy, Last et al. [48] found that the elastic modulus of the TM was higher in eyes with glaucoma than in normal eyes, suggesting an increased TM stiffness in eyes with glaucoma. Last et al. [48] also mathematically demonstrated that an increase in TM stiffness would theoretically lead to an increase in juxtacanalicular region flow resistance. These results indicated that changes in TM biomechanics might be involved in IOP elevation and glaucoma development [45–48]. Additionally, Gabelt et al. [49] showed that a reduction in cellularity and loss of TM cells in glaucomatous eyes resulted in a reduced outflow capacity and an increased IOP. The degeneration of the trabecular cells might cause more serious changes, including fusion of the trabecular meshwork [50]. A decreased TM thickness might be related to the increased outflow resistance and elevated IOP in glaucomatous eyes. Perhaps we can try to use the TM thickness evaluate the function of TM *in vivo*.

This study had several limitations. First, the majority patients with POAG were not visiting our clinic for the first time and had been using one or more antiglaucomatous drugs to lower IOP. Therefore, IOP was higher than 21 mmHg in only 46 eyes. Additionally, the use of the antiglaucomatous agents might have affected SC and the TM and the effects of these drugs were not evaluated. Second, our study sample size was relatively small, which made it impossible to investigate SC and TM morphology at different POAG stages. Future research is needed with a larger group of patients. Third, we only obtained canal measurements from each patient once. It is well known that IOP fluctuated throughout the day and, because SC anatomy was influenced by IOP, the effects of circadian rhythms on our measurements were not evaluated. Fourth, it is possible that the ultrasound biomicroscopy axial resolution (25 μm) affected SC coronal diameter measurements, if the resolution is higher, in POAG eyes smaller coronal diameter may be detected and the actual coronal diameter may be smaller than the value in this study.

## Conclusions

In conclusion, SC and the TM can be noninvasively, dynamically, and continuously imaged *in vivo* using 80-MHz ultrasound biomicroscopy. These images allow SC and TM parameters to be evaluated. Using such measurements, we found that patients with POAG have a less observable SC, smaller SC diameter, and decreased TM thickness than normal individuals. In addition, both SC coronal diameter and TM thickness were correlated with IOP. These results confirm that outflow structures are visible on 80-MHz ultrasound biomicroscopy images and that there is more than one indicator of SC status. Additionally, TM thickness may be a useful clinical measure for evaluating physiologic TM changes in patients with POAG.

## Supporting Information

S1 STROBE Checklist STROBE_checklist_v4_combined_PlosMedicine. (DOCX)
